# Hepatocellular Carcinoma (HCC) Metastasis to the Diaphragm Muscle: A Systematic Review and Meta-Analysis of Case Reports

**DOI:** 10.3390/cancers16173076

**Published:** 2024-09-04

**Authors:** Janusz Kocjan, Mateusz Rydel, Mariusz Adamek

**Affiliations:** 1 Diaphragm Concept Academy, Private Clinic Centre Specializing in Treating of Diaphragm Disorders, 32-300 Olkusz, Poland; 2 Department of Thoracic Surgery, Faculty of Medicine with Dentistry Division, Medical University of Silesia, 40-055 Katowice, Poland; 3 Faculty of Health Sciences with Institute of Maritime and Tropical Medicine, Department of Radiology, Medical University of Gdansk, 80-210 Gdansk, Poland

**Keywords:** hepatocellular, HCC, diaphragm, metastasis

## Abstract

**Simple Summary:**

Hepatocellular carcinoma (HCC) stands as a primary malignancy of the liver and represents a significant global health burden, particularly in regions where chronic hepatitis B and C infections prevail. Despite advancements in diagnostic techniques and therapeutic strategies, HCC continues to pose formidable challenges in clinical management due to its aggressive nature, frequent late-stage diagnosis, and metastasis to other organs. Although the incidence of metastasis to the diaphragm is low, this condition is one of few poor prognostic factors for HCC, making the early detection of diaphragm muscle involvement important in both primary and recurrent stages. The purpose of our study was to address two questions: (1) What symptoms or symptom clusters are reported among HCC patients with metastases to the diaphragm? (2) What risk factors for diaphragm muscle metastasis are present in HCC patients? Based on our findings, we conclude that the presence of hepatitis B and the localization of HCC cells in superior liver segments, particularly in the 8th liver segment, should be considered during the diagnostic process. Each patient in the reports presented with different symptoms, making it impossible to identify the leading symptom.

**Abstract:**

The purpose of this study was to conduct a systematic review and meta-analysis of case reports presenting HCC spread to the diaphragm muscle and to determine possible risk factors for this condition. An extensive literature search was performed using the following electronic databases: MEDLINE, CINAHL, ScienceDirect, Google Scholar, and DOAJ. A total of 18 articles describing 27 hepatocellular carcinoma patients were included in this review. The presence of HCC cells in the superior liver segment is strongly associated with metastases to the diaphragm. Among the two types of diaphragm involvement by HCC cells, diaphragm infiltration occurs much more frequently than diaphragm adhesion. However, an HCC nodule in the 8th liver segment and a higher number of liver segments involved by HCC cells predispose patients to diaphragm adhesion. Hepatitis B is a risk factor for diaphragm metastases in recurrent HCC. The tumor diameter is not associated with HCC spread to the diaphragm muscle. We did not find specific symptoms reported by patients that could indicate HCC metastasis to the diaphragm muscle. The presence of hepatitis B and the localization of HCC cells in superior liver segments, especially in the 8th liver segment, should be take into consideration in the diagnostic process.

## 1. Introduction

Hepatocellular carcinoma (HCC) is the most prevalent primary tumor of the liver, accounting for over 800,000 new cases and over 700,000 deaths annually [1]. It is also ranked among the fifth most common causes of cancer worldwide [2]. The management of HCC includes a wide range of treatment modalities tailored to the disease stage and the patient’s overall health. Current therapeutic approaches encompass surgical resection and liver transplantation for early-stage HCC; transarterial chemoembolization (TACE) and radiofrequency ablation for intermediate-stage disease; and systemic therapies, such as tyrosine kinase inhibitors (e.g., sorafenib and lenvatinib) and immune checkpoint inhibitors, for advanced HCC [3]. In recent years, significant attention has been given to Traditional Chinese Medicine as an alternative treatment option. Studies have shown that herbal and phytochemical compounds, such as curcumin, ginsenosides, wogonin, glycyrrhizin, astragalosides, and baicalein, can inhibit cancer stem cell (CSC) proliferation—responsible for malignant tumorigenesis, recurrence, metastasis, and drug resistance—induce apoptosis, and enhance CSC sensitivity to conventional therapies [4]. Despite significant advancements in treatment modalities, HCC is frequently diagnosed late in its course due to the absence of symptoms in the early stages of the disease, with a five-year survival rate ranging from 8% to 18.8% [5,6]. Various prognostic factors, including treatment modalities, tumor characteristics, the presence of extrahepatic metastases, serum biomarker levels, patient factors, and the etiology of liver disease, can significantly influence patient survival. Recent studies have shown that the upregulation of microRNA-520c-3p (miR-520c-3p) by HBV plays a critical role in enhancing the migration and invasion capabilities of HCC cells, contributing to tumor metastasis and promoting a more aggressive cancer phenotype [7]. An important factor influencing the effectiveness of these treatments is the tumor immune microenvironment (TIME), which supports tumor growth and progression and contributes to resistance to conventional therapies. Wu et al. presented a noninvasive approach to evaluating the TIME and predicting outcomes in HCC. This approach uses advanced imaging techniques combined with machine learning algorithms to assess immune cell infiltration and other key features of the TIME without the need for invasive biopsy procedures. This method allows for a comprehensive profile of the immune landscape within the tumor, enabling more accurate predictions of patient outcomes and responses to therapy [8].

The high incidence of HCC metastasis to other organs presents a significant clinical challenge, often resulting in a poor prognosis for affected patients and decreasing survival to one year [9]. The most frequent HCC metastatic sites include the lungs and lymph nodes (over 50%), followed by bone (28%), and then the adrenal glands, peritoneum, and omentum (approximately 10% each) [10]. Metastasis to the diaphragm represents a rare (about 10% patients according to autopsy studies [11] and less than 1% according to radiology studies [6]), yet clinically significant route of disease dissemination, including diaphragmatic fibrous adhesion (DFA) or pathological diaphragmatic invasion (DI) [12]. The low incidence of cancer spread to skeletal muscle such as the diaphragm is a result of a fluctuating blood flow due to muscle contractions, which renders it unsuitable for tumor cell harboring and growth. Additionally, lactic acid produced by skeletal muscle, as well as proteases and other inhibitors contained in muscles, suppress tumor invasion and growth [13,14,15].

The diaphragm is a double dome-shaped primary breathing muscle that separates the thoracic and abdominal cavities. It is a thin skeletal muscle composed of both slow-twitch and fast-twitch muscle fibers, and it is organized into three main parts: the sternal portion, the costal portion, and the lumbar portion with crura. The diaphragm’s muscle structure is supported by a central tendon made of dense collagenous connective tissue, which provides strength and durability to withstand the constant mechanical stresses during breathing and serves as the point of insertion for all parts of the muscle fibers. Each hemidiaphragm receives motor innervation from the phrenic nerve, which is formed within the cervical plexus and contains fibers from the spinal roots C3–C5. The majority of the diaphragm’s vascular supply is delivered by the inferior phrenic arteries, which arise directly from the abdominal aorta, with venous blood draining via the phrenic veins [16].

Molecular and cellular pathways of diaphragm infiltration by HCC remain poorly described. Diaphragm involvement can occur via the falciform ligament (a fibrous band connecting the liver to the anterior abdominal wall and diaphragm) serving as a conduit for HCC cells to migrate from the liver to the diaphragm, or by a hematogenous spread through the inferior phrenic artery, which can supply tumors adhering to the diaphragm [17,18]. Similarly, little is known about the symptoms or groups of symptoms that may indicate the metastasis of HCC to the diaphragm muscle, raising difficulties in the early recognition of further tumor progression.

The literature on the subject contains several original research papers on diaphragm metastases during the course of hepatocellular carcinoma, grouped into one systematic review. These studies focus on comparing the short- and long-term surgical outcomes of these patients without taking into account the diagnostic aspects of this clinical condition. However, in the existing literature, we can also find a few papers describing single cases of HCC invasion to the diaphragm with detailed patient symptom presentations. To the best of our knowledge, to date, there is no systematic review of case studies and case reports on this condition. Therefore, to fill the gap and shed new light on the intricacies of metastatic HCC involving the diaphragm, in the present paper, we collect, analyze, and summarize the diagnostic aspects of diaphragm metastasis during the course of HCC.

## 2. Materials and Methods

This systematic review was conducted according to the Preferred Reporting Items for Systematic Reviews and Meta-Analyses (PRISMA) statement [19]. The ethical review was not required for this type of study.

### 2.1. Search Strategy

An extensive literature search was performed using the following electronical databases: MEDLINE, Cumulative Index to Nursing and Allied Health Literature (CINAHL), Science Direct, Google Scholar, and Directory Open Access Journal (DOAJ) from inception to 31 April 2024. Furthermore, one additional search was conducted on 10 June 2024 to ensure completeness. We used the following terms: “hepatocellular carcinoma” and “HCC” to identify articles relevant for this review. The search terms were combined with “diaphragm”, “hemidiaphragm”, or “diaphragm metastasis” in order to obtain the greatest possible number of literature positions. We did not impose demographic restrictions. However, we restricted the language of keywords to English.

### 2.2. Study Selection and Criteria

From the obtained search results, we selected papers published as case studies or case reports in peer-reviewed scientific journals. The titles, publication dates, and authors’ names were reviewed to exclude duplicate articles involving the repetition of cases. Next, all selected studies were screened to determine whether they fulfilled the following inclusion criteria: HCC diagnosis with metastasis to the diaphragm muscle confirmed by imaging findings, studies confined to human subjects, and patient age > 18 years. We discarded articles that did not fit the topic or whose full texts were inaccessible. In the final literature review, we manually scanned the reference lists of each article qualified for review, looking for other relevant studies. The screening process of eligible articles was conducted by a team of two authors (JK and MR). All data items were verified by a third author (MA). Any disagreements were discussed and resolved by consensus.

### 2.3. Data Evaluation

The critical appraisal checklist for case reports developed by the Joanna Briggs Institute (JBI) was used to assess the quality of the presented studies and the risk of bias [20]. This questionnaire consists of 8 questions, each with three possible answers scored on a 2-point scale: 100 points (answer: “yes”) and 0 points (answers: “no” or “unclear”). The overall score is the sum of the obtained points divided by 8. The final score is grouped into three categories: (I) low risk of bias—studies that met at least 75% of the quality criteria; (II) moderate risk of bias—studies in the range between 50% and 74% of the quality criteria; and (III) high risk of bias—studies that met less than 50% of the quality criteria.

We excluded papers that scored below 50% and were characterized by a high risk of bias from the final literature review.

### 2.4. Data Extraction

From each paper, we extracted the following sociodemographic and clinical data for our analysis: age, gender, ethnicity, type of diaphragm involvement (infiltration, adhesion), side of diaphragm involvement, symptoms, laboratory blood test results, tumor size, tumor localization (lobe, section, and segment), number of liver segments involved, and time since previous hepatectomy in recurrent cases.

### 2.5. Clinical Definitions

#### 2.5.1. Tumor Diameter

The tumor size was defined as the longest diameter of the cancer mass. For further assessment, patients with HCC were separated into groups according to tumor size as follows: 0.1–2.0, 2.1–5.0, 5.1–10.0, and 10.1–20.0 cm.

#### 2.5.2. HCC Metastasis to the Diaphragm

Two types of diaphragm metastasis were recognized in this study. Diaphragm infiltration was defined as HCC with pathological diaphragm invasion, while diaphragm adhesion was determined as HCC fibrous adhesion without pathological diaphragm invasion. The term “diaphragm involvement” encompasses either diaphragm infiltration or diaphragm adhesion.

#### 2.5.3. Primary and Recurrent HCC

A primary HCC was defined as the first occurrence of tumor cells growing, progressing, and forming a cancerous mass in the liver, with or without metastasis to other organs. Recurrent HCC was defined as intrahepatic or extrahepatic HCC cells that reappeared after previous treatment and a period of remission.

### 2.6. Data Synthesis

Initially, we assumed excluding papers with missing or incomplete data. However, each paper was characterized by an individual level of collected data, restricting the study to patients with complete datasets, resulting in a small sample size. We considered setting a cutoff value to exclude papers when missing patient data exceeded 50%, but this could introduce selection bias. Therefore, we decided to use all available data from the included papers. In the results chapter, we provide the exact number of patients analyzed for each factor.

### 2.7. Data Processing

In some studies, the laboratory tests results were presented as exact values, while in others, they were given as qualitative data (e.g., normal, decreased, and increased levels). This created interpretation challenges. To maximize the population size, we transformed the available quantitative data into qualitative data, presenting a broader clinical picture than using numerous precise data points.

### 2.8. Data Analysis

All data were collected and organized using Microsoft Excel (Microsoft Corp, Seattle, WA, USA). Raw data were summarized using descriptive statistics, with means and standard deviations for continuous variables and frequencies and percentages for dichotomous variables. All statistical tests were two-sided and a *p*-value < 0.05 indicated statistical significance in this study. A statistical analysis was performed using PQSTAT software version 1.8.6.102.

## 3. Results

### 3.1. Study Selection

**Figure 1:**
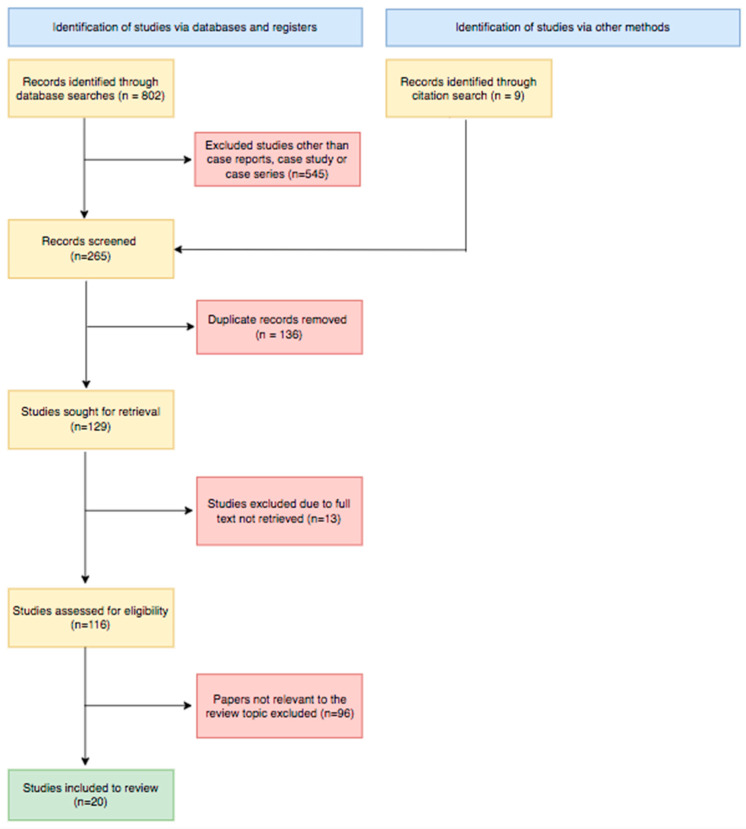
Flowchart of article selection process.

Initially, we retrieved a total of 5891 records from five databases (604 from MEDLINE, 35 from CINAHL, 21 from ScienceDirect, 51 from Directory Open Access Journal, and 91 from Google Scholar). After a thorough selection process that takes into account the inclusion criteria, the article’s compliance with the topic of our study, the availability of the full text, and the exclusion of duplicated papers, 17 articles were included in the final stage of evaluation [21,22,23,24,25,26,27,28,29,30,31,32,33,34,35,36,37]. Additionally, from reference list checking during screening, we added two case reports [38,39] and one article [40], which is a cohort study consisting of nine patients where all clinical data were presented individually, as in case reports. Overall, 20 articles were settled on to review. The flow diagram of the full article selection process is illustrated in Figure 1.

### 3.2. Results of Risk of Bias Analysis

**Figure 2:**
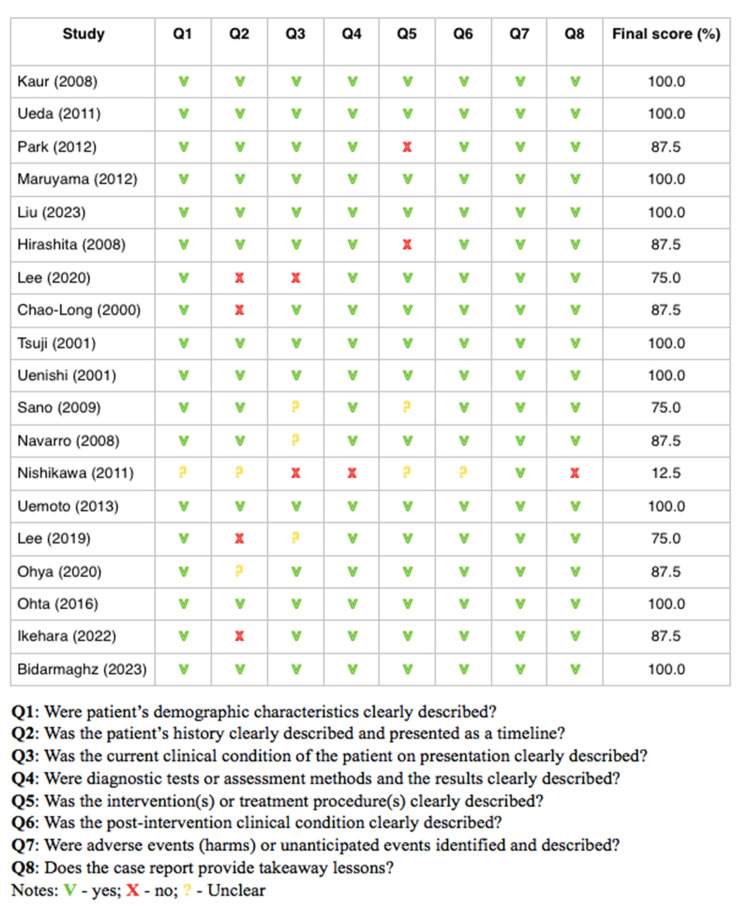
Quality assessment of analyses studies.

One study was denied at the base of the low score (12.5%) obtained in the JBI scale due to a poor paper quality and high risk of bias [21]. The remaining papers [22,23,24,25,26,27,28,29,30,31,32,33,34,35,36,37,38,39] obtained a score equal to or over 75%, which characterized them as having a low risk of bias. Similar results were demonstrated in refer to Kim et al.’s study [40]. Thus, a total of 19 articles were implemented in the review. Details about the quality assessment of each study are presented in Figure 2.

### 3.3. General Patients Characteristics

Eighteen case reports and one cohort study described 27 hepatocellular cancer patients with metastasis to the diaphragm muscle aged between 35 and 82 years with a mean age of the sample of 59.29 (standard deviation = 12.46). The majority of HCC cases were in the age group of 51–60 years old (37%; N = 10)—compared to other groups: 31–40 y/o (11.1%, N = 3), 41–50 y/o (11.1%, N = 3), 61–70 y/o (18.5%; N = 5), 71–80 y/o (18.5%; N = 5), and 81–90 y/o (3.7%; N = 1).

Of the 27 patients’ cases, the vast majority were males (96.3%, N = 26) and represent an Asian population (92.6%, N = 25), except two subjects who came from Africa and Australia. Detailed demographic characteristics were as follow: 37% South Korean (N = 10), 37% Japanese (N = 10), 11.1% Chinese (N = 3), and 3.7% (N = 1) Malaysian and Taiwanese. None of the studies provided the patients’ anthropometric parameters such as height, weight, and BMI index.

### 3.4. Clinical Characteristics of Patients

Table 1 presents the tumor characteristics for patients with HCC involving the diaphragm.

In total, 40.7% (n = 11; age: mean = 60.81, SD = 8.78, range: 47–75) of patients were diagnosed with primary HCC with metastasis to the diaphragm muscle, while 59.3% had recurrent tumors (n = 16; age: mean = 58.25, SD = 14.66, range: 35–82; time from hepatic lobectomy: mean = 12.5, SD = 9.88, range: 6–30 months). There was no age (*p* = 0.608) differences between the groups.

The mean tumor size was significantly greater in the primary than recurrent patients group (10.84 ± 2.23 vs. 5.56 ± 3.45, *p* < 0.001). The mean number of liver segments involved by HCC cells was higher (*p* = 0.153) in the primary HCC group (2.44 ± 1.48) than recurrent HCC group (1.42 ± 1.08).

The superior segments (I, II, IVa, VII, and VIII) of the liver were much more often occupied by HCC nodules than inferior segments (III, IVb, V, and VI). No differences were found between these two liver parts in terms of the tumor diameter (6.12 ± 3.15 vs. 5.20 ± 2.77, *p* = 0.474) and number of segments involved (1.22 ± 0.44 vs. 1.00 ± 0.00, *p* = 0.176). There were no differences between primary and recurrent HCC in terms of nodule localization for lobes, sections, and segments.

Twenty-one patients (77.7%) had HCC metastases exclusively to the diaphragm. The remaining six patients (22.3%) also had metastases to other organs, including the right atrium (n = 1), colon (n = 2), stomach (n = 1), and intercostal muscles (n = 1), lung (n = 1).

### 3.5. Symptoms Presentation

In Kim et al.’s cohort study, the patients’ symptoms were not described. In three cases, the patients were asymptomatic and the diagnosis of metastasis was made during a routine medical examination or during a follow-up examination after a hepatectomy. Out of the remaining 15 cases, 3 of them were hospitalized due to a tumor rupture. As this condition is a potentially life-threatening complication of HCC situation characterized by other specific symptoms, these patients were excluded from the symptomatological analysis.

Among the remaining 12 patients, we did not find a leading symptom or symptoms cluster that could indicate the metastasis of HCC to the diaphragm muscle, both for primary HCC, as well as recurrent HCC. The remaining symptoms reported by individual patients were as follows: fever, abdominal pain, loss of appetite, painful swelling in the epigastric region, ribs pain, loss of weight, fatigue, flank pain, and chest pain. One of the patients reported a painless nodule during abdominal palpation. Respiratory failure was not described in any of the case reports. In one paper, Park et al. stated a clear patient breath sound during a medical examination [24].

### 3.6. Liver Condition Laboratory Blood Tests

**Figure 3:**
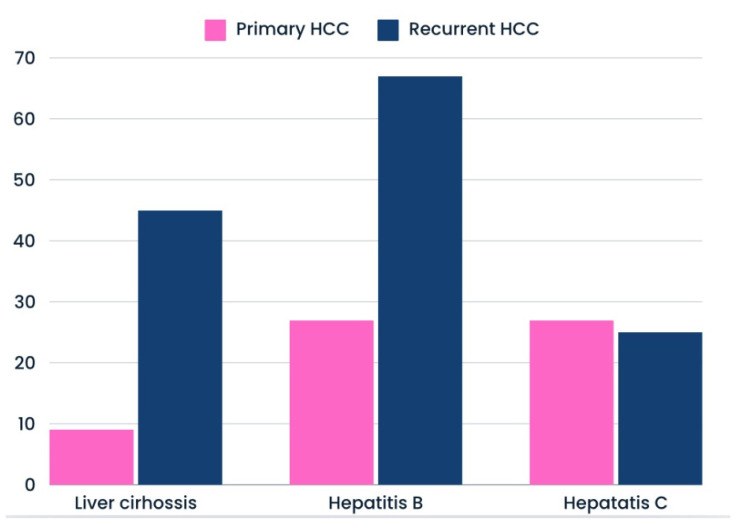
Percentage of patients with liver disease during primary and recurrent HCC course with diaphragm involvement.

The most common diagnosed liver disease was hepatitis B (56%; N = 14), followed by liver cirrhosis (36%; N = 9) and hepatitis C (28%; N = 7). These first two disease entities were significantly more common among patients with HCC recurrence than the primary tumor. The differences were not observed in the case of HCV. Detailed data are available in Figure 3.

By analyzing the overall data, it can be seen that most patients had elevated values of liver condition laboratory blood tests: AlAT (75%, N = 6/8), AspAT (62.5%, N = 5/8), ALP (55.5%, N = 5/9), Billirubin total (57.4%, N = 4/7), and GGTP (60%, N = 3/5). Similar findings were found in reference to the tumor markers AFP (71%, N = 17/24) and PIVKA II (66.6%, N = 10/15). However, all subjects (N = 9) obtained class A in a Child–Pugh scale. In Table 2, we present the laboratory blood test results with a division of primary and recurrent HCC.

### 3.7. Diaphragm Muscle Metastasis

Among metastases to the diaphragm, infiltration predominates much more often (85.1%, n = 23) than adhesion (14.9%, n = 4). There was no age (57.95 ± 11.99 vs. 67.0 + 14.07, *p* = 0.185) or tumor size (6.66 ± 3.85 vs. 8.85 + 5.54, *p* = 0.468) differences between the groups.

The mean number of liver segments involved during the HCC process was significantly greater in the case of diaphragm adhesion than infiltration (3.50 ± 1.29 vs. 1.36 ± 0.95, *p* < 0.001). In all four diaphragm adhesion cases, the HCC nodule involved a minimum of two liver segments and a sixth liver segment was always engaged.

The left hemidiaphragm was affected by HCC metastasis in 48% (N = 12) of subjects, while 52% (N = 13) had the right hemidiaphragm involved. In 100% of analyzed cases, the tumor located on left lobe of the liver was associated with metastasis to the left hemidiaphragm. In the case of the right liver lobe, the tumor spread to the right hemidiaphragm in 13 situations, while in 6 situations it spread to the left hemidiaphragm from the following segments: S4 (n = 4), S1 (n = 1), and S6 (n = 1).

Only in one study was the elevation of the diaphragm noted, while in the remaining, the radiological assessment of the diaphragm was normal.

### 3.8. Other Factors

Only in one case was HCC present at margin resection. Similar findings were found in reference to lymph nodes metastasis. In four out of six cases, an HCC vascular invasion was noted. Most cases concerned the portal vein rather than the hepatic vein. Interestingly, all cases with vascular invasion were located in the right liver lobe, while cases without angioinvasion were found in the left lobe.

### 3.9. Survival Prognosis

All patients underwent the surgical resection of the tumor. In 16 studies, a postoperative observation was described. In 93.8% (n = 15) of surgery, the postoperative course was described as uneventful, while in one case, it was described as good. The average postoperative hospital stay length was 10.12 +5.96 days. In 12 studies, long term follow-up was performed. Most of the patients (n = 9, 75%) were alive when the case report was published. At the base of these findings, the 6-month survival rate was 83.3% (10 out 12 patients), while the 1-year and 2-year survival rates were 50% (6 out 12 patients) and 25% (3 out 12 patients), respectively. The survival rates for primary and recurrent HCC did not differ at 6 months.

## 4. Discussion

Hepatocellular carcinoma (HCC) is the most common primary liver tumor known for its aggressive character. Advanced diagnostic modalities and current available treatment methods have improved the patient survival rate, but have also increased the identification of extrahepatic metastases of HCC as a result. Although the incidence of metastasis to the diaphragm is low, this state is one of few poor prognostic factors for HCC, so it is clinically important to detect diaphragm muscle involvement in primary and recurrence stages early. In this review, we synthesized existing knowledge from available case reports to provide new insights into symptoms and clinical variables during diaphragm involvement in HCC patients.

HCC symptoms differ from those of other cancers because subjects do not experience disease-specific symptoms or physical dysfunctions until the condition becomes severe and advanced. The most prevalent symptoms are fatigue, a lack of energy, stomach or abdominal pain/distension, a loss of appetite, change in taste, sleep disturbance, and distress [41]. In the present paper, we attempted to identify a characteristic symptom or set of symptoms that could indicate the presence of diaphragm metastases. However, each patient in the reports presented with different symptoms, making it impossible to determine the leading symptoms. Despite the involvement of the diaphragm muscle, none of the analyzed studies reported respiratory problems in the patients. This is likely due to the topography of cancer metastases, which were located only within one dome of the diaphragm. It appears that the area of muscle infiltration was too small to cause respiratory problems, similar to cases of unilateral diaphragmatic paralysis or phrenic nerve palsy, where respiratory symptoms are also absent or mild. However, respiratory problems are visible in cases of diaphragmatic hernia due to thermal damage after radiofrequency ablation for the treatment of unresectable primary and secondary HCC malignancies [42,43]. This underscores the insidious nature of HCC malignancy and suggests that metastases to the diaphragm may occur without respiratory impairment. However, their presence may signal more serious issues and necessitate imaging tests.

Liver cirrhosis and hepatitis B are the major risk factors for the development of this type of liver cancer [44]. In our work, we noted a significantly higher rate of liver cirrhosis and HCV type B in patients with diaphragm metastases from recurrent HCC compared to the first disease. Therefore, it seems that both liver diseases are risk factors for diaphragm metastases during HCC recurrence. These factors appear to have no effect on diaphragmatic metastases in individuals diagnosed with HCC for the first time.

Most patients had elevated values of liver function laboratory tests, but 25–45% had them within the normal range, even in cases of disease recurrence. This allows us to conclude that normal liver results in a blood test do not exclude the absence of metastases. Interestingly, in some cases, the normal AFP value was noted despite recurrent disease. This might suggest a new HCC cell line that is different from the primary tumor.

Arizumi showed that large HCCs are more susceptible to the invasion of local blood vessels, are more likely to develop metastases, and satellite foci are often found at the time of diagnosis [45]. Miyagawa et al. demonstrated that a large tumor diameter is a significant factor contributing to multiorgan invasion in extrahepatic HCC [46]. Our results add new knowledge in this field, but contradict previous findings for three reasons. First, the average size of the tumor in the presented case reports of patients with HCC metastasis to the diaphragm was 6.88 cm, and the percentage of patients with a tumor larger than 10 cm was small (only 20% of patients). These results are similar to data from other scientific publications on patients with HCC without any metastases [47]. Secondly, we observed metastases to the diaphragm more frequently when HCC cells were located in the right liver lobe. However, there was no difference in tumor diameter between the right and left lobes. Finally, despite diaphragm infiltration representing a more frequent type of diaphragm involvement than diaphragm adhesion during the course of HCC, we again observed no differences in the tumor diameter. This suggests that factors other than the tumor size lie at the base of diaphragmatic metastases.

The segmental distribution of HCC according to Couinaud’s division showed that segments 4, 7, and 8 represented the most common locations (27.4%). These findings are consistent with the theory of liver segmental volumes, based on portal branching and perfusion, reported in the literature, where these three segments are characterized by the highest total liver volume [48]. However, given that segment 2 was one of the next most frequently affected segments in our study, this calls into question these theories regarding the diaphragm, because the second segment has a smaller volume than segments 3 and 5, which were less often invaded. Therefore, it seems that the position of the lobes in relation to the diaphragm may be important in this case. Superior liver segments directly bordering the diaphragm muscle were more frequently invaded by HCC cells than inferior segments, with no differences in the tumor size between these two liver regions. This emphasizes the necessity of a later diaphragm muscle assessment by practitioners when superior liver segments are involved by HCC cells.

Most patients had nodules in only one segment of the liver. However, patients with adhesion had a greater number of involved segments than patients with infiltration. This result is surprising because it might seem that the more occupied the segments, the greater the risk of infiltration. All patients with adhesion also had nodules located in the s8 segments, making them risk factors for this type of diaphragm involvement.

Several advanced imaging techniques are used to detect and evaluate diaphragm involvement, including Computed Tomography (CT), Magnetic Resonance Imaging (MRI), Positron Emission Tomography (PET), Ultrasound (US), and chest X-ray. Among these, the first three methods seem to be the most useful. In CT scans, particularly contrast-enhanced ones, diaphragmatic metastasis may appear as soft tissue masses or the thickening of the diaphragm, sometimes with irregular enhancement patterns. MRI provides a superior contrast resolution compared to CT, making it better suited for identifying smaller metastases or those with complex anatomical relationships. PET scans may reveal areas of the increased uptake of the radiotracer (usually fluorodeoxyglucose, FDG), indicating active tumor tissue in the diaphragm muscle. The utility of ultrasound and chest radiography is limited. While they cannot directly identify diaphragm invasion by the tumor, they can sometimes detect abnormalities such as the elevation or deformation of the diaphragm if the metastatic lesion is large, prompting further investigation with more advanced imaging. When necessary, biopsies are performed to confirm the diagnosis histologically.

The precise differentiation between diaphragm invasion and diaphragm fibrous adhesion in the course of HCC is challenging. Imaging techniques like CT and MRI are particularly valuable for making a preoperative diagnosis. On CT scans, diaphragm adhesion may present as a close apposition of the tumor to the diaphragm without disrupting the diaphragm’s integrity, with no signs of the tumor extending into or through it. In contrast, diaphragm invasion may be indicated by the irregular thickening or disruption of the diaphragm, with the tumor appearing to breach the diaphragm and showing signs of a direct extension into the diaphragmatic muscle or even through it. A mass protruding into the chest cavity or adjacent structures can also suggest invasion. In MRI exams, particularly in T1-weighted images, adhesion may appear as a hypointense line that remains uninterrupted by the tumor, indicating that the diaphragm’s integrity is preserved. Invasion, on the other hand, is typically characterized by the loss of the normal diaphragmatic contour and changes in the signal intensity within the diaphragm. The tumor may show continuity between the liver mass and an abnormal diaphragmatic mass, suggesting direct invasion. When imaging is inconclusive, a direct visualization and palpation during surgery can help differentiate between invasion and adhesion. Invasion involves the diaphragm muscle, while adhesion is characterized by the formation of fibrous bands attaching the liver to the diaphragm. In ambiguous cases where tissue samples are available, a histopathological examination can confirm the presence of tumor cells within the diaphragm muscle (infiltration) versus fibrous tissue attachment without invasion (adhesion) [49].

The surgical management of HCC with metastasis to the diaphragm is complex and requires a multidisciplinary approach to optimize outcomes and manage associated risks. Although diaphragmatic involvement is serious, it may still be amenable to aggressive surgical resection, particularly if it is isolated and the patient is in a good condition. This makes it a potentially treatable condition if detected early. The cornerstone of successful HCC surgery is achieving complete tumor resection with clear surgical margins while preserving liver function and minimizing postoperative complications. When the diaphragm is involved, obtaining negative surgical margins (R0 resection) can be challenging due to the close anatomical relationship between the diaphragm and liver. Incomplete resection or positive margins (R1 resection) can lead to higher recurrence rates and poorer outcomes. Not all patients with HCC and diaphragm metastasis are suitable for surgery due to the high-risk nature of the procedure. A careful preoperative assessment, including an evaluation of the liver function, overall health, and tumor burden, is crucial to determine if the benefits of surgery outweigh the risks. Given the aggressive nature of HCC with diaphragm metastasis, surgery alone may not be sufficient. A multimodal approach, including adjuvant therapies such as chemotherapy, targeted therapy, or radiotherapy, may be necessary to manage the residual disease and reduce the risk of recurrence [50].

According to the American Joint Committee on Cancer (AJCC)’s 8th staging system, HCC with diaphragm involvement is classified as an advanced-stage disease with a poor prognosis [51]. However, some studies have suggested that diaphragm involvement does not necessarily indicate an unfavorable outlook [52,53,54,55]. The mortality rate of HCC patients, although improving, still appears to be extraordinarily high. HCC metastasis to the diaphragm muscle tends to have a somewhat better prognosis compared to other common metastatic sites, such as the lungs or bones, which often signal more a widespread systemic disease. This can lead to complications like respiratory distress or pathological fractures. The localized nature of diaphragm metastasis can sometimes allow for more targeted interventions, potentially improving outcomes compared to a more extensive systemic spread. The five-year survival rate for patients with HCC and lung metastases typically ranges between 10% and 20% [56,57,58,59]. In contrast, the survival rates for HCC with bone metastasis are generally very low, often less than 10% [60,61,62]. At this stage, treatment tends to be less effective due to the diffuse nature of the disease and is generally supportive rather than curative.

Our study has some limitations. First of all, there is a large disproportion between the number of women and men, as well as the over-representation of the Asian population. Due to this fact, our results refer mainly to men of Asian origin and should be cautiously implemented in other geographical areas and in the female population. The presented work is also limited by an incomplete dataset for several characteristics in individual papers. It should also be taken into account that despite the comprehensive search, for some papers, only an abstract was available, which may exclude other valuable papers from the analysis. Finally, although selected papers were characterized by a high quality in the JBI scale, the case reports provide a non-randomized sample and are at the lowest level of the modern medical evidence hierarchy. These four points raise a need to perform further research in this area.

## 5. Conclusions

Based on the results presented in our work, several conclusions can be drawn that could have not been reached from previous papers:

1. Among diaphragm involvement by HCC cells, the diaphragm invasion is more frequently observed than diaphragm adhesion.
2. The presence of HCC nodules in segment 6 is a risk factor for diaphragm adhesion.
3. A greater number of affected liver segments in the course of HCC contributes more to diaphragm adhesion than to diaphragm infiltration.
4. Metastases to the diaphragm muscle occurred much more often when the superior segments of the liver were involved by HCC nodules than the lower ones. The presence of HCC nodules in the upper segments also seems to be a risk factor for diaphragmatic metastases. This indicates the need to monitor patients at a later stage.
5. There are no specific symptoms reported by patients that could indicate HCC metastases to the diaphragm muscle. Comparing our data with other available scientific works, the presence of respiratory problems may indicate more serious problems such as a diaphragmatic hernia.
6. Tumor size is not associated with HCC spread to diaphragm muscle, while hepatitis B is the risk factor for diaphragmatic metastases in patients diagnosed with HCC for the second time.

## Figures and Tables

**Table 1:** Tumor characteristics.

Variables	M ± SD (Range: Min–Max)/Number of Subjects (Percentage of All)
**Largest tumor diameter:**	
mean	6.88 ± 3.92 (1.7–15.0)
<2 cm	2 (10.0%)
2–5 cm	6 (30.0%)
5–10 cm	8 (40.0%)
>10 cm	4 (20.0%)
**HCC nodule localization (liver lobe):**	
Right	18 (75.0%)
Left	6 (25.0%)
**HCC nodule localization (liver sections):**	
right posterior	6 (25.0%)
left lateral	6 (25.0%)
left medial	4 (16.6%)
right anterior	1 (4.2%)
mixed	7 (29.2%)
**HCC nodule localization according to Coinaud classification:**	
S1	1 (4.55%)
S2	4 (18.2%)
S3	3 (13.6%)
S4	7 (31.8%)
S5	3 (13.6%)
S6	5 (22.7%)
S7	7 (31.8%)
S8	7 (31.8%)
**Number of liver segments involved by HCC:**	
1	15 (68.2%)
2	4 (18.2%)
3	1 (4.55%)
4	1 (4.55%)
5	2 (9.1%)
mean	1.81 ± 1.28 (1–5)
**Edmondson-Steiner grading system:**	
Grade I (well-differentiated)	1 (5.9%)
Grade II (moderately differentiated)	11 (64.7%)
Grade III (poorly differentiated)	5 (29.4%)
Grade IV (undifferentiated)	0 (0.0%)

**Table 2:** Number of patients with normal and elevated results of laboratory blood test among patients with primary and recurrent HCC with metastasis to the diaphragm muscle.

Variables		Primary	Recurrent
**AlAT (n = 8)**	Normal	3	0
	Elevated	4	1
**AspAT (n = 8)**	Normal	3	0
	Elevated	4	1
**ALP (n = 9)**	Normal	3	0
	Elevated	4	2
**Billirubin total (n = 7)**	Normal	3	0
	Elevated	3	1
**GGTP (n = 5)**	Normal	2	0
	Elevated	3	0
**AFP (n = 24)**	Normal	2	5
	Elevated	7	10
**PIVKA II (n = 15)**	Normal	1	4
	Elevated	2	8

## Data Availability

The data presented in this study are available upon request from the corresponding author.
